# NSUN2 alleviates doxorubicin-induced myocardial injury through Nrf2-mediated antioxidant stress

**DOI:** 10.1038/s41420-022-01294-w

**Published:** 2023-02-04

**Authors:** Yi Wang, Yuxin Zan, Yingying Huang, Xiaoyun Peng, Shinan Ma, Ji Ren, Xiao Li, Lin Wei, Xiaoli Wang, Yahong Yuan, Junming Tang, Zhongqun Zhan, Zhixiao Wang, Yan Ding

**Affiliations:** 1 Hubei Key Laboratory of Embryonic Stem Cell Research, Taihe Hospital, Hubei University of Medicine, 442000 Shiyan, Hubei China; 2 Cardiovascular Department, Yiyang People’s Hospital, 413000 Yiyang, Hunan China; 3 Department of Cardiology, University of Chinese Academy of Sciences-Shenzhen Hospital, 518107 Shenzhen, China; 4 Cardiovascular Department, Taihe Hospital, Hubei University of Medicine, 442000 Shiyan, Hubei China; 5 Hubei Clinical Research Center for Umbilical cord blood hematopoietic stem cells, Taihe Hospital, Hubei University of Medicine, 442000 Shiyan, Hubei China

**Keywords:** Experimental models of disease, Mechanisms of disease

## Abstract

Doxorubicin (DOX) is a commonly used antitumor drug, but its application has been limited because of its strong cardiac damage. This study aims to explore the role of NSUN2 in DOX-induced heart injury. C57BL/6J mice were intraperitoneally injected with 20 mg/Kg DOX to induce heart injury. After 3 days, the cardiac function, cardiac histopathology, myocardial apoptosis, and the expression level of NSUN2 were detected. In vitro, H9C2 cells were transfected with NSUN2 siRNA or overexpressed lentivirus and then treated with 500 ng/ml DOX. After 24 h, the changes in reactive oxygen species (ROS), apoptosis, and NSUN2 expression were detected. After DOX treatment, both in vitro and in vivo experiments showed that the cardiac function decreased, the number of apoptotic cells increased, and the expression level of NSUN2 increased. Interfering the expression of NSUN2 by siRNA promoted DOX-induced heart injury, while overexpression of NSUN2 could inhibit DOX-induced heart injury. Further study showed that NSUN2 promoted antioxidative stress by upregulating the Nrf2 protein level. In addition, NSUN2 overexpression could increase the half-life of Nrf2 mRNA. m5C RNA methylation immunoprecipitation (MeRIP) also showed that the level of Nrf2 m5C mRNA was significantly increased in NSUN2 overexpressed group when compared to the GFP group. NSUN2 enhances the expression of Nrf2 by promoting Nrf2 mRNA m5C modification and enhances its antioxidative stress effect to alleviate DOX-induced myocardial injury.

## Introduction

Doxorubicin (DOX) is an antitumor chemotherapeutic agent widely used in clinical practice [1–3], but its cardiotoxicity can lead to tumor treatment failure or even death. Statistics from recent years show that the incidence and mortality of cardiac toxicity are much more serious than we expected [4].

At present, the mechanisms of DOX-induced myocardial injury mainly include (1) oxidative stress injury induced by excessive reactive oxygen species (ROS) activation, (2) topoisomerase II binding to cardiomyocyte TOP2β to damage DNA, and (3) ROS damage mitochondria [5]. DOX can produce excessive reactive ROS through enzymatic or non-enzymatic pathways. In addition, anthraquinone doxorubicin can also produce large amounts of ROS under the action of reductase and dehydrogenase [6]. Excessive ROS accumulation could damage mitochondria and other cell energy factories, thereby causing cell apoptosis. It can also activate the body’s oxidative stress system, stimulating inflammatory cells to release inflammatory factors, such as IL-1, TNF, and NF-κB. This, in turn, promotes damage and apoptosis of cardiomyocytes and ultimately causes heart damage, structural changes, and functional decline.

NSUN2 is an RNA methyltransferase. It was first discovered as a tRNA methyltransferase, but later studies found that lncRNA, mRNA, and miRNA could all be methylated by NSUN2. Furthermore, NSUN2 could also promote the generation and expression of m5C in mammalian mitochondria, and play an important role in cell proliferation and growth [7, 8]. The purpose of this study was to investigate the role of NSUN2 in DOX-induced heart injury.

## Materials and methods

### Animal feeding and model building

All animals were kept on 12 h light/dark cycle in a temperature-controlled room with ad libitum access to food and water. The use of animals and all animal protocols were approved by the Institutional Animal Care and Use Committee (IACUC) of the Hubei University of Medicine. Hundred healthy male C57BL/6J mice aged 8–10 weeks (weighted 23–25 g) were purchased from the Animal Center of the Hubei University of Medicine. Mice were divided into saline group (NS), DOX group, shNC + saline group (shNC NS), shNC + DOX group (shNC DOX), shNSUN2 + saline group (shNSUN2 NS), shNSUN2 + DOX group (shNSUN2 DOX), control vector + saline group (control NS), control vector + DOX group (control DOX), NSUN2 vector + saline group (NSUN2 NS), and NSUN2 vector + DOX group (NSUN2 DOX). shNC group, shNUSN2 group, control group and NSUN2 group were tail-vein injected with 5 × 10^11^vg corresponding AAV9 virus, respectively. Four weeks later, the four groups of mice were randomly divided into saline group (NS) and doxorubicin group (DOX). NS group received an intraperitoneal injection of 0.9% saline, while DOX group received 20 mg/kg (diluted to 2 mg/ml with 0.9% saline) DOX.

### Ultrasonic collection of small animals

Three days after 0.9% saline or 20 mg/kg DOX treatment, mice were anesthetized with 1.0–2.0% isoflurane. After the respiration of mice was stable and the heart rate was about 400 beats per minute, B-mode and M-mode ultrasound images of the left ventricular long-axis section, left ventricular short-axis section, and four-chamber heart section were collected by Vevo 2000 MS-400 probe. The model maker and experimenter were independent of each other, and the experimenter was unaware of the model grouping.

### Establishment of DOX-induced injury model in H9C2 cells

H9C2 cells were donated by Dr. He [9], and the cells in the logarithmic growth phase were plated into 6-well plates. After the cell density reached more than 95%, 0.5 mg/ml DOX or 0.9% saline was added to the medium respectively for 24 h.

### NSUN2 siRNA transfection

H9C2 cells in the logarithmic growth phase were plated into 6-well plates. When the cells reached 40–50% fusion, 50 nM siNC or siNSUN2 mixture was transfected with lipo 3000 kit according to the operation instructions. After 48 h, cells were collected, and the total RNA or protein was extracted. The siNSUN2 sequences are presented in Table 1.

**Table 1:** The sequence of NSUN2 siRNA and Nrf2 primers.

Name	Sequence
NSUN2 siRNA-1	GGAAGAAATGGACTACCTT
NSUN2 siRNA-2	CTGAAGTACGAACCAGATT
NSUN2 siRNA-3	GAAGATGAAGGTCATTAAC
Nrf2 Forward	5′ CAGCATGATGGACTTGGAATTG 3′
Nrf2 Reverse	5′ GCAAGCGACTCATGGTCATC 3′
GAPDH- Forward	5′ GCATCTTCTTGTGCAGTGCC 3′
GAPDH- Reverse	5′ GATGGTGATGGGTTTCCCGT 3′

### NSUN2 overexpression lentivirus transfection

H9C2 cells in the logarithmic growth stage were seeded into 6-well plates. When the cell density reached 60%, 2 μl GFP or GFP-NSUN2 lentivirus solution (WZ Biosciences) was added into the cell medium, respectively. Twenty-four hours later, 2 μg/ml puromycin was added to screen NSUN2 stably overexpressed cells.

### TUNEL detection

Cell apoptosis was detected by TUNEL kit (Beyotime, C1090), and the operation process was carried out according to the instruction. After dyeing, 1μl DAPI staining solution (Beyotime, C1005) was added and reacted for 5–10 min at room temperature, and then the dead cells were observed and photographed with a fluorescent microscope.

### Immunohistochemistry

The heart tissues fixed with 4% paraformaldehyde were sectioned continuously with a thickness of 5 μm, and the slices were treated according to our previous method [10]. Rabbit NSUN2 (Proteintech, 20854-1-AP, 1:200) and Nrf2 (Proteintech, 16396-1-AP, 1:200) polyclonal antibodies were added and incubated at 37 °C for 2 h, and then the sections were incubated with goat anti-rabbit second antibody (ZSGO-BIO, PV-9001) at room temperature for 1 h. Finally, the results were tested by using the DAB Chromogenic Kit (ZSGO-BIO, ZLI-9018) according to the operating instructions.

### ROS detection

Reactive oxygen species were detected using the ROS kit (Beyotime, S0033S) according to operating instructions. The cells were observed under a microscope and photographed.

### Real-time quantitative PCR (RT-PCR)

Total RNA was extracted by Trizol (Beyotime, R0016). The mRNA expression level of Nrf2 was detected by SYBR Green PCR kit (Beyotime, D7260). The primers were presented in Table 1.

### Actinomycin D-treated cells

The control group and NSUN2 overexpressed H9C2 cells in the logarithmic growth phase were seeded into 12-well plates. When the cell density reached 95%, 2 µg/ml of actinomycin D was added for 0, 4, 8, 12, 16, and 24 h, respectively. Then the expression level of Nrf2 was detected by RT-PCR, and the half-life times were calculated.

### Western blot

The cells were lysed with RIPA (Beyotime, P0013B) containing 1% PMSF (Beyotime, ST505). The protein concentrations were determined using BCA kit (Beyotime, P0010S) according to the instructions, followed by sodium dodecyl sulfate-polyacrylamide gel electrophoresis (SDS-PAGE) at 20 mg/well. After the electrophoresis, the proteins were transferred onto the NC membrane, and rabbit NSUN2 (Proteintech, 20854-1-AP, 1:200), Nrf2 (Proteintech, 16396-1-AP, 1:200), Bax (Beyotime, AF0057, 1:500), Bcl-2 (Beyotime, AB112, 1:500), HO-1 (Beyotime, AF1333, 1:500), NQO1 (Beyotime, AF7614, 1:500), and α-Tubulin Rabbit polyclonal antibody (Beyotime, AF5012, 1:500) were incubated overnight at 4 °C. Then Horseradish peroxidase (HRP) labeled goat anti-rabbit IgG (H + L) (Beyotime, A0208, 1:2000) was added and incubated at room temperature for 1 h. Finally, the concentration of the proteins was measured using the enhanced chemiluminescence (ECL) chemiluminescence kit (Beyotime, P0018).

### m5C MeRIP

The total RNA of H9C2 cells was extracted with Trizol and divided into IgG and m5C groups, and 7 µl of IgG or m5C antibody was added to the two groups respectively, followed by incubating overnight at 4 °C. The next day, 50 µl magnetic beads were added, and the mixtures were incubated in a shaking table at 4 °C for 6 h. After centrifuging at 12,000 rpm at 4 °C for 2 min, the supernatant was discarded, and 1 ml RIPA containing 1% PMSF was added. Then, the RNA was extracted with Trizol, and the level of m5C Nrf2 mRNA was detected by RT-PCR.

### Dot blot

Total RNA was diluted to 200, 400, 600, and 800 ng/μl with DEPC water and denatured at 95 °C for 10 min. One microliter of denatured RNA was transferred onto NC films, then the NC films were cross-linked with ultraviolet rays at 500 J for 2 min. The remaining steps were the same as Western blot.

### Statistical analysis

Graphpad Prism version 9.0.0 and SPSS 25.0 were used to analyze the data. All the data were shown as mean ± standard deviation, and the samples were in normal distribution. The Student’s *t*-test was used for comparisons between the two groups. The survival curve was tested by Kaplan–Meier. *P* < 0.05 was considered as statistical significance.

## Results

### The effects of DOX treatment on cardiac function and NSUN2 expression

**Fig. 1:**
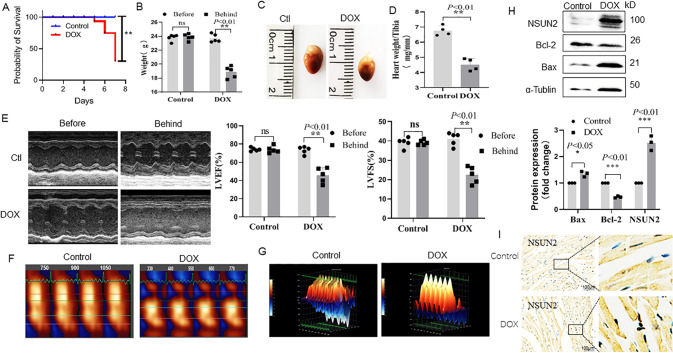
Effects of DOX treatment on cardiac function and the expression level of NSUN2 in myocardial cells. **A** The survival rate of mice treated with DOX (*n* = 5). **B** The weights of mice treated with DOX for 3 days (*n* = 5). **C** Macroscopic observation of mouse heart after 3 days of DOX treatment. **D** Effect of DOX on the ratio of heart weight to tibia in mice (*n* = 4). **E** M-mode ultrasonic image and LVEF%, LVFS% statistical chart of short-axis section of mouse heart before and after DOX treatment (*n* = 5). **F**, **G** Two-dimensional speckle tracking technology displayed the diastolic phase (red) and systolic phase (blue) of the wall motion monitored by each tracer point shown in the figure. **H** Western blot was used to detect the expression of apoptosis-related proteins and NSUN2 protein in the heart tissues of two groups (*n* = 3). **I** Immunohistochemistry was used to detect the expression of NSUN2 in the myocardium of DOX treatment group and saline group (Bar = 100 µm). The survival rate was analyzed by survival curve, the measurement data were shown as mean ± standard deviation, and compared by Student’s *t*-test. *P < 0.05; **P < 0.01; ***P < 0.001.

First, mice were intraperitoneally injected with DOX at 15, 20, and 25 mg/Kg d. The results showed that mice treated with 15 mg/kg DOX had no significant cardiac function changes for 7 days (data not shown), and 53% of mice in the 25 mg/kg group died within 7 days (Fig. 1A), while mice in the 20 mg/kg group had decreased cardiac function and low mortality. Therefore, in subsequent experiments, 20 mg/kg was selected as the DOX acute myocardial injury model.

In this study, the results showed that after DOX treatment for 3 days, the weight of mice decreased significantly (Fig. 1B). Both heart volume (Fig. 1C) and heart weight (Fig. 1D) decreased significantly. In addition, echocardiography showed a significant reduction in LVEF and LVFS in the DOX-treated group (Fig. 1E), and two-dimensional spot tracking showed abnormal wall movements in DOX-treated mice (Fig. 1F, G). In addition, we also found that the expression of Bcl-2 in the DOX group was significantly reduced, while Bax was significantly increased (Fig. 1H). The expression of NSUN2 in the DOX group was significantly higher than that in the NS group (Fig. 1H, I).

### NSUN2 gene interference promotes DOX-induced myocardial injury

**Fig. 2:**
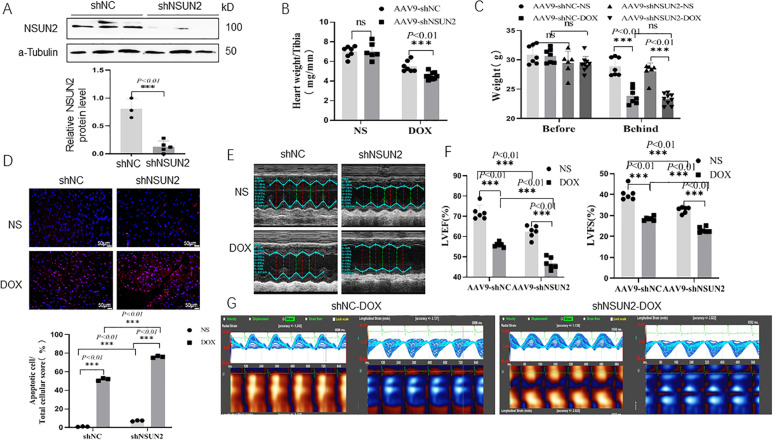
Interfering with NSUN2 expression promoted DOX-induced myocardial injury. **A** Western blot was used to detect the expression of NSUN2 protein in the heart tissue of mice treated with DOX (*n* = 3). **B**, **C** The weight and heart weight of mice in each group (*n* = 7). **D** TUNEL detected myocardial cell apoptosis in four groups of mice (*n* = 3, Bar = 50 µm). **E** M-mode ultrasonic representation of short-axis section. **F** Statistical chart of LVEF% and LVFS% of four groups of mice detected by small-animal ultrasound (*n* = 6). **G** Two-dimensional speckle tracking technology displays the diastolic phase (red) and systolic phase (blue) of the wall motion monitored by each tracer point in the order shown in the figure. The measurement data were shown as mean ± standard deviation and compared by Student’s *t*-test. *P < 0.05; **P < 0.01; ***P < 0.001.

shNSUN2 AAV9 was intravenously injected to interfere with the expression of NSUN2 in cardiomyocytes. Four weeks later, the DOX-induced myocardial injury model was established by intraperitoneal injection. The results demonstrated that the expression of NSUN2 in the heart tissue significantly decreased after injection of shNSUN2 AAV9 (Fig. 2A). When compared with the shNC group, the heart weight (Fig. 2B), body weight (Fig. 2C), the number of apoptotic cells (Fig. 2D), the LVEV and LVFS (Fig. 2E, F) decreased more obviously. Two-dimensional speckle tracking showed that compared with the shNC group, DOX-induced abnormal wall motion in the shNSUN2 group was more serious (Fig. 2G). These results suggested that NSUN2 interference promotes DOX-induced heart injury.

### NSUN2 overexpression inhibits DOX-induced myocardial injury

**Fig. 3:**
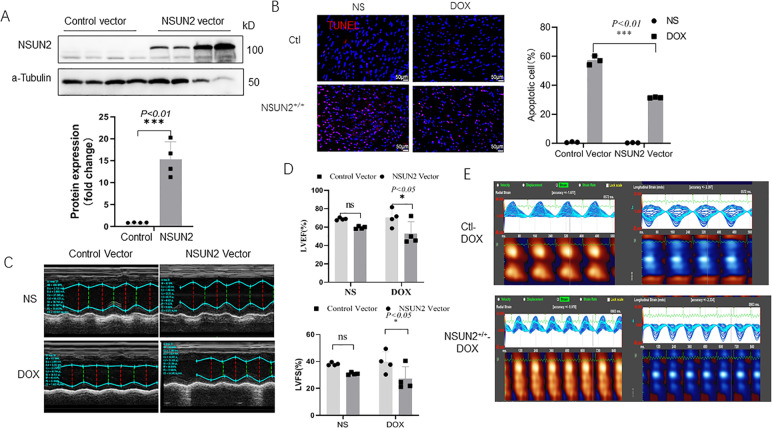
Ectopic expression of human NSUN2 in vivo alleviated DOX-induced myocardial injury. **A** Western blot detected the expression of NSUN2 protein in heart tissue after 4 weeks of NSUN2 or control AAV9 treatment (*n* = 4). **B** TUNEL detected the myocardial cell apoptosis in four groups of mice (*n* = 3, Bar = 50 µm). **C** M-mode ultrasonic representation of short-axis section. **D** Statistical chart of LVEF% and LVFS% of four groups of mice detected by small-animal ultrasound (*n* = 4). **E** Two-dimensional speckle tracking technology displays the diastolic phase (red) and systolic phase (blue) of the wall motion monitored by each tracer point in the order shown in the figure. The measurement data were shown as mean ± standard deviation and compared by Student’s *t*-test. *P < 0.05; **P < 0.01; ***P < 0.001.

Human NSUN2 AAV9 was injected intravenously to make it ectopic expressed in mouse cardiomyocytes. Four weeks later, DOX myocardial injury model was established. The results showed that after NSUN2 AAV9 injection, the expression of NSUN2 in myocardial cells increased significantly (Fig. 3A). When compared with the control group, the NSUN2 group had fewer apoptotic cells (Fig. 3B) and higher LVEV and LVFS (Fig. 3C, D). Two-dimensional speckle tracking showed that the abnormal wall motion induced by DOX in the NSUN2 group was significantly lighter than that in the AAV9-GFP group (Fig. 3E).

### NSUN2 protects H9C2 cardiomyocytes from DOX-induced injury

**Fig. 4:**
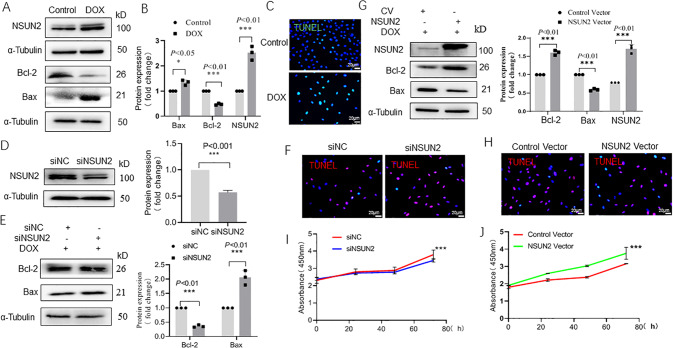
NSUN2 alleviates the injury of myocardial cells induced by DOX. **A**, **B** Western blot detected the protein level of apoptosis gene Bcl-2 and Bax, as well as NUSN2 (*n* = 3). **C** TUNEL detected the apoptosis of H9C2 cells after DOX treatment (*n* = 3, Bar = 20 µm). **D** Western blot detected the transfection efficiency of siNSUN2 in H9C2 cells (*n* = 3); **E** Western blot detected the effect of interfering of NSUN2 on apoptosis-related proteins in H9C2 cells after DOX treatment (*n* = 3). **F** TUNEL detected the effect of NSUN2 interference on DOX-induced apoptosis (*n* = 3, Bar = 20 µm). **G** Western blot detected the efficiency of NSUN2 overexpression in H9C2 cells. **H** TUNEL detected the effect of NSUN2 overexpression on DOX-induced H9C2 cell apoptosis (*n* = 3, Bar = 20 µm). **I**, **J** Effects of interference and overexpression of NSUN2 on the proliferation of H9C2 cells. The measurement data were shown as mean ± standard deviation and compared by Student’s *t*-test. *P < 0.05; **P < 0.01; ***P < 0.001.

After H9C2 cells were treated with 500 ng/ml DOX for 24 h, the protein expression levels of Bax, Bcl-2, and NSUN2 in H9C2 cells were detected by Western blot. When compared with the control group, the expression level of Bax and NSUN2 in the DOX group increased significantly (*P* < 0.01, Fig. 4A, B), but Bcl-2 decreased significantly (*P* < 0.01). TUNEL staining showed that the number of apoptotic cells in the DOX group was significantly higher than that in the control group (Fig. 4C). After siRNA transfection, the protein level of NSUN2 decreased obviously (*P* < 0.01, Fig. 4D). After DOX treatment, the Bax level in siNSUN2 group was significantly higher (*P* < 0.05) and Bcl-2 was significantly lower (*P* < 0.01, Fig. 4E) than those of siNC group. TUNEL staining showed that the number of apoptotic cells in the siNSUN2 group was larger than that in the siNC group (Fig. 4F). After being infected with NSUN2 lentivirus, the expression level of NSUN2 in H9C2 cells increased significantly (Fig.4G). After DOX treatment, it was found that compared with the empty vector group, the expression of Bax in the NSUN2 group decreased while Bcl-2 increased significantly (*P* < 0.01, Fig. 4G). We also observed a decrease in the number of apoptotic cells (Fig. 4H). MTT test indicated that the cell proliferation rate decreased in the siNSUN2 group (Fig.4I) but increased in the NSUN2 overexpression group (Fig. 4J) significantly (*P* < 0.01).

### NSUN2 promotes the antioxidant stress effect of cardiomyocytes by upregulating Nrf2

**Fig. 5:**
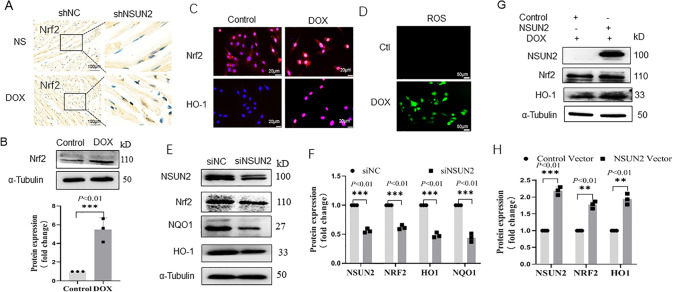
NSUN2 upregulates Nrf2-mediated antioxidant stress in myocardial cells in DOX-induced myocardial injury. **A**, **B** Immunohistochemistry and western blot detected the expression level of Nrf2 in myocardial cells with and without DOX treatment (Bar = 100 µm). **C** IF detected the expression of Nrf2 and HO-1 after DOX-induced H9C2 cell injury (Bar = 20 µm). **D** IF detected the changes in intracellular ROS after DOX treatment. **E**, **F** Western blot detected the expression level of Nrf2 and HO-1 (*n* = 3, Bar = 20 µm) after NSUN2 interference in H9C2 cells. **G**, **H** Western blot detected the effect of NSUN2 overexpression on the expression of Nrf2 and HO-1 in H9C2 cells injured by DOX. The measurement data were shown as mean ± standard deviation and compared by Student’s *t*-test. *P < 0.05; **P < 0.01; ***P < 0.001.

Immunohistochemical detection showed that the level of Nrf2 in DOX-treated cardiomyocytes was higher than that in the NS group. After interfering with shNSUN2, the expression of Nrf2 significantly decreased than that in the shNC group (Fig. 5A). After DOX treatment, the protein level of Nrf2 and HO-1 in H9C2 cells was significantly higher than that in the NS group (*P* < 0.01, Fig. 5B, C). DCFH-DA probe detection showed that the level of ROS in the DOX group was significantly higher than that in the NS group (Fig. 5D). Compared to the control group, Nrf2 and downstream NQO1 and HO-1 were significantly decreased in the siNSUN2 group (Fig. 5E, F, *P* < 0.01), while significantly increased in NSUN2 group (Fig. 5G, H, *P* < 0.01).

### NSUN2 increases the level of Nrf2 mRNA m5C modification to promote its stability

**Fig. 6:**
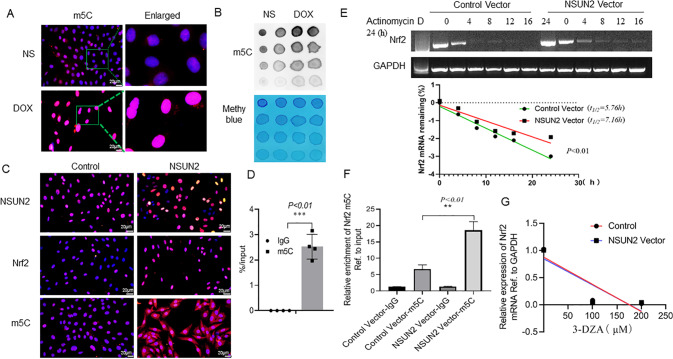
NSUN2 promotes Nrf2 mRNA m5C methylation modification in H9C2 cells. **A** IF detected the intracellular m5C level of H9C2 cells after DOX treatment (*n* = 3, Bar = 20 μm). **B** Dot blot detected the changes of m5C methylation in H9C2 cells after DOX treatment. **C** IF detected the effect of NSUN2 overexpression on NSUN2 and Nrf2 expression levels and m5C enrichment in H9C2 cells (Bar = 20 μm). **D** m5C MeRIP-PCR detected the effect of NSUN2 overexpression on the enrichment of m5C in H9C2 cells (*n* = 4). **E** RT-PCR detected the effect of NSUN2 overexpression on the half-life of Nrf2 mRNA (*n* = 3). **F** m5C MeRIP test the content of m5C modified Nrf2 mRNA in NSUN2 and GFP group. **G** RT-PCR detected the effect of NSUN2 overexpression on the half-life of Nrf2 mRNA when treated with methylase inhibitor 3-DZA (*n* = 3). The measurement data was showed as mean ± standard deviation, and compared by student’s t-test. **P < 0.05; **P < 0.01; ***P < 0.001.

In order to make clear how NSUN2 regulates Nrf2, we first analyzed whether there is an interaction between NSUN2 and Nrf2 proteins by co-immunoprecipitation, and the results showed that there was no interaction between them (data not shown). To further make clear whether NSUN2 acts as an RNA methyltransferase, we detected the m5C content of H9C2 cells after DOX treatment. Immunofluorescence (Fig. 6A) and dot blot (Fig. 6B) showed that the m5C level in the DOX group was significantly higher than that in the NS group. After transfection of the NSUN2 virus, the expression of NSUN2 increased, and in addition, the levels of Nrf2 and m5C increased significantly (Fig. 6C). m5C MeRIP test also confirmed the existence of m5C modified Nrf2 mRNA (Fig. 6D). Then, we treated both control and NSUN2 group H9C2 cells with actinomycin D (2 ng/ml) to inhibit mRNA synthesis, and detected the Nrf2 mRNA levels of the two group cells after 0, 2, 4, 8, 12, 16, and 24 h. The results showed that the half-life of Nrf2 mRNA in the NSUN2 group was significantly higher than that of the control group (Fig. 6E). These results indicated that NSUN2 promoted the stability of Nrf2 mRNA. m5C MeRIP test also confirmed that the content of m5C modified Nrf2 mRNA in the NSUN2 group was higher than that of the GFP group (Fig. 6F). After treatment with 3-Deazaadenosine (3-DZA) to inhibit methylation, it was found that there was no significant change in Nrf2 mRNA level in GFP-NSUN2 compared with GFP group (Fig. 6G).

### NSUN2 participates in the composition of mouse heart structure

**Fig. 7:**
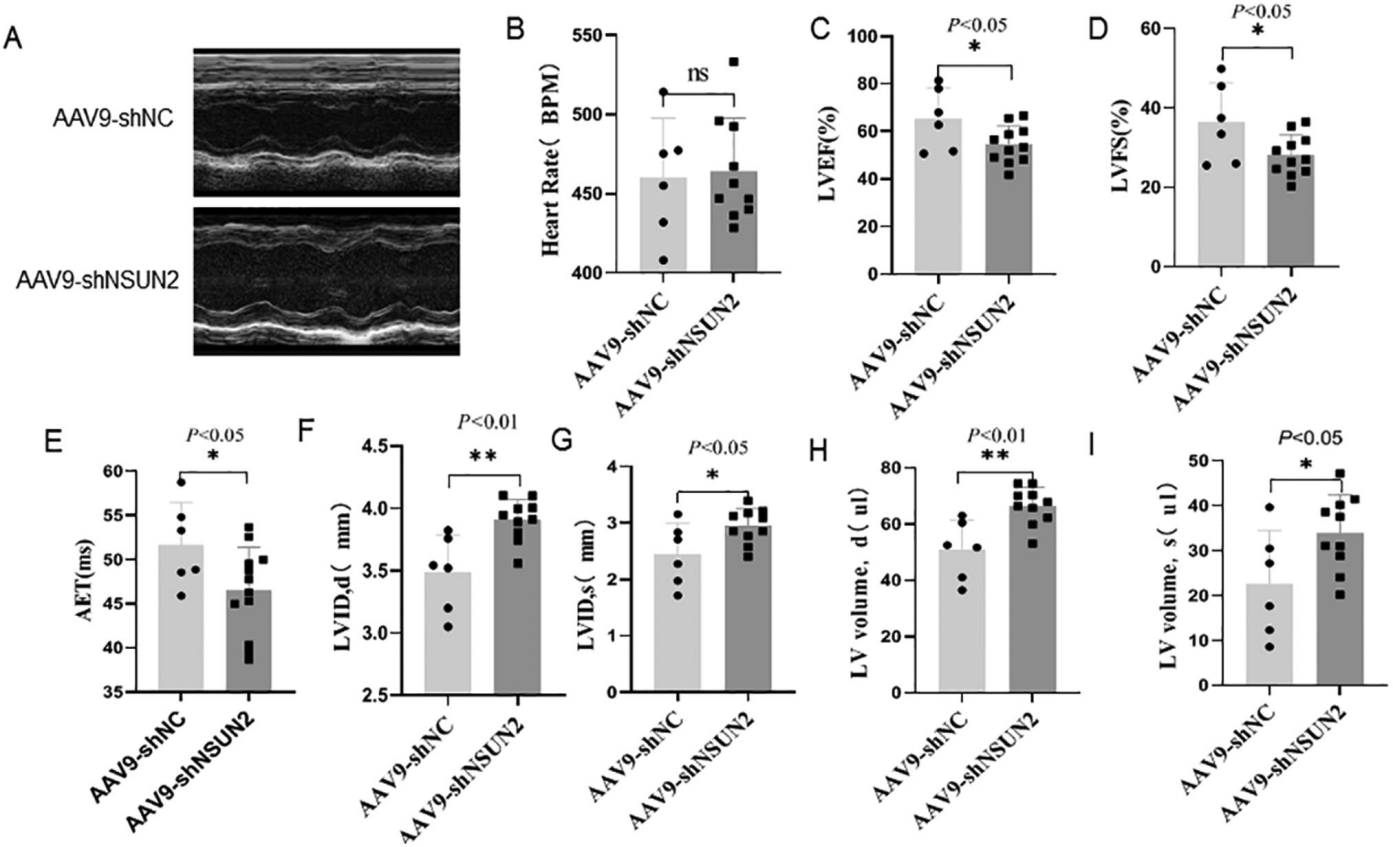
NSUN2 is involved in the composition of mouse heart structure. **A** Representative pictures of M-mode ultrasound in short-axis section of shNC (*n* = 6) and shNSUN2 mice (*n* = 10). **B**–**I** There is no statistical difference between the two groups of mice in BPM chart, **C**–**I** AET, LVEF%, LVFS%, LVIDD, LVIDS, LVed Vol, LVEVVOL, statistical chart of two groups of mice. The measurement data were shown as mean ± standard deviation and compared by Student’s *t*-test. *P < 0.05; **P < 0.01.

To clarify the role of NSUN2 in the development of the heart, small-animal ultrasonic detection was performed 4 weeks after the injection of shNC or shNSUN2 AAV9 virus (Fig. 7A). The results showed that there was no significant difference in heart rate between the two groups (Fig. 7B). When compared with the shNC group, the values of LVEF% (Fig. 7C), LVFS% (Fig. 7D), and aortic ejection time (AET, Fig. 7E) in shNSUN2 group decreased significantly (*P* < 0.05), while the diastolic diameter of the left ventricle (LVIDD, Fig. 7F), left ventricular systolic inner diameter (LVIDS, Fig. 7G), left ventricular systolic volume (LVed Vol, Fig. 7H), and left ventricular diastolic volume (LVes Vol, Fig. 7I) increased significantly (*P* < 0.01). These results indicated that inhibiting the expression of NSUN2 could thicken the ventricular wall, increase the left ventricular volume, shorten the aortic ejection time, and decrease the systolic function in mice. However, NSUN2 overexpression had no significant effect on cardiac function (Fig. S1). These results suggest that NSUN2 plays an important role in maintaining normal cardiac function.

**Fig. 8:**
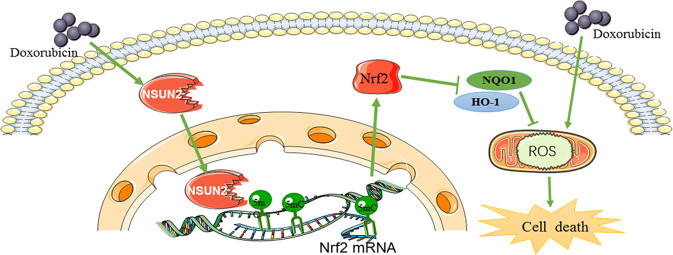
Scheme of NSUN2 in DOX-induced myocardial injury. NSUN2 promote the methylation modification of Nrf2 mRNA, and increase the level of Nrf2 protein, which activates the downstream antioxidant stress response and reduces the damage caused by ROS.

In conclusion, these results showed that DOX treatment activates cells to produce a large amount of ROS, which damages cells. At the same time, it also activates the cell defense mechanisms to upregulate the expression of NSUN2, which promotes the methylation modification of Nrf2 mRNA and increases the level of Nrf2 protein. Finally, the elevated Nrf2 further activates the downstream antioxidant stress response and reduces the damage caused by ROS (Fig. 8).

## Discussion

When DOX acts on the heart, it causes inflammatory cell infiltration, Ca^2+^ overload, energy metabolism disorder, and so on. All these pathophysiological changes can promote cells to produce excessive ROS, and it is too late for the body to eliminate excessive ROS [5]. Excessive ROS can activate the body’s oxidative stress reaction, which can lead to myocardial cell necrosis and apoptosis [11]. How can we effectively remove ROS without damaging other functions of the body? Studies have shown that activating the body’s own antioxidant stress system to resist oxidative stress may be a new therapeutic strategy.

NSUN2 can methylate all kinds of coding RNA and promote or inhibit the stability of target mRNA in cell growth. Studies have shown that [12] NSUN2-mediated mRNA methylation regulates the levels of p27 and CDK1 in the process of replicative aging. In addition, it was also found that NSUN2 could enhance the translation of intercellular adhesion molecule 1 (ICAM-1) and protect vascular endothelium from inflammation [13]. To sum up, NSUN2-catalyzed mRNA m5C modification will lead to different or even completely opposite results. The potential mechanism needs further study.

In this study, our results showed that the expression level of NSUN2 increased in the DOX-induced myocardial injury model, and the cardiac function damage and myocardium cell apoptosis induced by DOX increased, while the cardiac function damage and myocardium cell apoptosis induced by DOX decreased when NSUN2 was overexpressed. These results indicated that NSUN2 could inhibit DOX-induced heart injury.

As ROS plays a very important role in DOX-induced heart injury, we examined the effect of NSUN2 on ROS and found that NSUN2 could inhibit DOX-induced ROS production. Nrf2 controls the expression of a series of antioxidant genes and is the core factor in regulating antioxidant stress [14]. It has been proved that Nrf2 can enhance the antioxidant capacity of cells by upregulating the expression of detoxification enzyme genes such as NAD(P)H, quinone oxidoreductase (NQO1), and heme oxidase-1(HO-1) [15, 16]. The protective effect of Nrf2 on the heart has also been confirmed in many animal models, including uncompensated cardiac remodeling and cardiac dysfunction caused by aortic arch constriction (TAC), ventricular remodeling, and cardiac dysfunction caused by myocardial infarction, etc.

In this study, our results showed that the level of Nrf2 increased 3 days after DOX injection, which indicated that the oxidative stress reaction in the heart was activated. Recent studies have also shown that when treated with DOX, the expression of Nrf2 in myocardial tissue increased in a time-dependent manner, and the expression of downstream genes such as HO-1 and NQO1 were upregulated [14]. In addition, DOX decreased the expression of Keap1, and the redox state of Keap1 protein affected by DOX-induced oxidative stress [17]. However, this slight transient upregulation of Nrf2 was not enough to overcome the oxidative stress injury induced by DOX. Therefore, since the degree and duration of Nrf2 upregulation are limited, the protective effect is limited. So, if we find a way to prolong the action time of Nrf2 or promote its expression, we can fight DOX-induced myocardial injury. It has been reported that the upregulation of Keap1/Nrf2/ARE signaling pathway induced by N-chlorosuccinimide (NCs) was an effective treatment for myocardial protection from DOX toxicity [18]. In this study, our results showed that NSUN2 could upregulate the expression of Nrf2 protein in DOX-induced myocardial injury model.

Our further study indicated that both in vitro and in vivo, NSUN2 could promote the expression of the Nrf2 protein. The mechanism may be through promoting m5C methylation modification of Nrf2 mRNA and prolonging the half-life of Nrf2 mRNA. The upregulated Nrf2 protein could improve the cell’s ability to resist oxidative stress, and resist DOX-induced oxidative stress injury.

To sum up, this study found that NSUN2 could activate the body’s own antioxidant stress response and inhibit the DOX-induced myocardial injury through Nrf2 m5C methylation modification. NUSN2 participated in the composition of cardiac structure and promoted the proliferation of myocardial cells.

## Supplementary information

Supplementary Figure Legend

Original Data File

Figure S1

## Data Availability

All the data used during the study are available from the corresponding author on request.
