# Correction to: NSUN2 alleviates doxorubicin-induced myocardial injury through Nrf2-mediated antioxidant stress

**DOI:** 10.1038/s41420-023-01377-2

**Published:** 2023-03-27

**Authors:** Yi Wang, Yuxin Zan, Yingying Huang, Xiaoyun Peng, Shinan Ma, Ji Ren, Xiao Li, Lin Wei, Xiaoli Wang, Yahong Yuan, Junming Tang, Zhongqun Zhan, Zhixiao Wang, Yan Ding

**Affiliations:** 1 Hubei Key Laboratory of Embryonic Stem Cell Research, Taihe Hospital, Hubei University of Medicine, 442000 Shiyan, Hubei China; 2 Cardiovascular Department, Yiyang People’s Hospital, 413000 Yiyang, Hunan China; 3 Department of Cardiology, University of Chinese Academy of Sciences-Shenzhen Hospital, 518107 Shenzhen, China; 4 Cardiovascular Department, Taihe Hospital, Hubei University of Medicine, 442000 Shiyan, Hubei China; 5 Hubei Clinical Research Center for Umbilical cord blood hematopoietic stem cells, Taihe Hospital, Hubei University of Medicine, 442000 Shiyan, Hubei China

**Keywords:** Experimental models of disease, Mechanisms of disease

Correction to: *Cell Death Discovery* 10.1038/s41420-022-01294-w, published online 04 February 2023

The original version of this article contained errors in the author affiliations. The correct affiliations can be found below. The original article has been corrected.

Yi Wang^1,2,6^, Yuxin Zan^1,6^, Yingying Huang^1,6^, Xiaoyun Peng^1,6^, Shinan Ma^1^, Ji Ren^1^, Xiao Li^1^, Lin Wei^1^, Xiaoli Wang^1^, Yahong Yuan^1^, Junming Tang^1^, Zhongqun Zhan^3^, Zhixiao Wang^1,4^, Yan Ding^1,5^

Aff 1 Hubei Key Laboratory of Embryonic Stem Cell Research, Taihe Hospital, Hubei University of Medicine, Shiyan, Hubei 442000, China

Aff 2 Cardiovascular Department, Yiyang people’s hospital, Yiyang, Hunan, 413000

Aff 3 Department of Cardiology, University of Chinese Academy of Sciences-Shenzhen Hospital, Shenzhen 518107, China

Aff 4 Cardiovascular Department, Taihe Hospital, Hubei University of Medicine, Shiyan, Hubei 442000, China

Aff 5 Hubei Clinical Research Center for Umbilical cord blood hematopoietic stem cells, Taihe Hospital, Hubei University of Medicine, Shiyan 442000, Hubei, China

6 These authors contributed equally

Corresponding Authors:

Yan Ding, E-mail: 20090990@hbmu.edu.cn;

Zhixiao Wang, E-mail: zhixiaowang@whu.edu.cn;

Zhan Zhongqun, E-mail: zzqun21@yahoo.com.cn

