# Supplementary material for: NSUN2 alleviates doxorubicin-induced myocardial injury through Nrf2-mediated antioxidant stress
Source: Cell Death Discov. 2023 Feb 4;9:43. doi: 10.1038/s41420-022-01294-w (PMC9899217; doi:10.1038/s41420-022-01294-w)
Supplement: Supplementary file 1 — Supplementary Figure Legend [file 41420_2022_1294_MOESM1_ESM.docx]

Fig.S1. NSUN2 overexpression has not significantly effect on mice heart function. A: representative pictures of M-mode ultrasound in short-axis section of control and NSUN2 mice. B-I: There is no statistical difference between the two groups of mice in BPM chart, C-I: AET, LVEF%, LVFS%, LVIDD, LVIDS, LVed Vol, LVEVVOL, statistical chart of two groups of mice (P>0.05, n=6). The measurement data was showed as mean ± standard deviation, and compared by student’s t-test.
