# Supplementary figures and images for: NSUN2 alleviates doxorubicin-induced myocardial injury through Nrf2-mediated antioxidant stress
Source: Cell Death Discov. 2023 Feb 4;9:43. doi: 10.1038/s41420-022-01294-w (PMC9899217; doi:10.1038/s41420-022-01294-w)

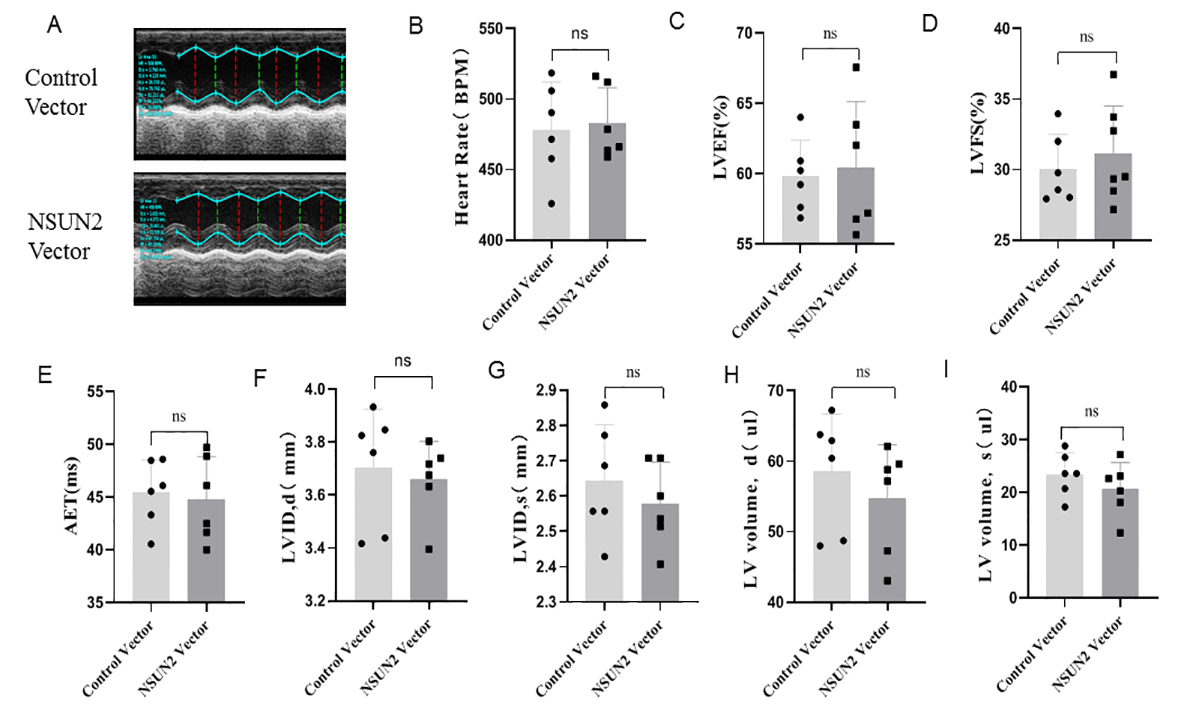

Supplement: Supplementary file 3 — Figure S1 [file 41420_2022_1294_MOESM3_ESM.tif]
