# Supplementary material for: NSUN2 alleviates doxorubicin-induced myocardial injury through Nrf2-mediated antioxidant stress
Source: Cell Death Discov. 2023 Feb 4;9:43. doi: 10.1038/s41420-022-01294-w (PMC9899217; doi:10.1038/s41420-022-01294-w)
Supplement: Supplementary file 2 — Original Data File [file 41420_2022_1294_MOESM2_ESM.docx]

Figure 1 H

Ctl DOX

a-tubulin

Ctl DOX

NSUN2

Ctl DOX

Bax

Ctl DOX

Bcl-2

Figure 2A	shNC	shNSUN2

α-Tublin

shNC	shNSUN2

NSUN2

Figure 3A	Control	NSUN2

α-Tublin

Control	NSUN2

NSUN2

Figure 4 A

Ctl DOX

α-Tublin

Ctl DOX

NSUN2

Ctl DOX

α-Tublin

Ctl DOX

Bax

Ctl DOX

Bcl-2

Figure 4 D	siNC siNSUN2

α-Tublin

siNC siNSUN2

NSUN2

Figure 4 E

siNC siNSUN2

Bcl-2

siNC siNSUN2

α-Tubuli

siNC siNSUN2

Bax

Figure 4 G	CV NSUN2+/+

α-Tublin

CV NSUN2+/+

Bax

CV NSUN2+/+

Bcl-2

Figure 5B	Ctl DOX

α-Tublin

Ctl DOX

Nrf2

Figure 5E	siNC siNSUN2

NSUN2

siNC siNSUN2

α-Tublin

siNC siNSUN2

Nrf2

siNC siNSUN2

HO1

siNC siNSUN2

NQO1

Figure 5G

CV NSUN2+/+

α-Tublin

CV NSUN2+/+

NSUN2

CV NSUN2+/+

Nrf2

CV NSUN2+/+

HO1
